# Assessing caseworker perceptions of client engagement in Danish child and family welfare

**DOI:** 10.3389/fsoc.2025.1591213

**Published:** 2025-10-31

**Authors:** Maiken Pontoppidan, Signe Boe Rayce

**Affiliations:** VIVE—The Danish Center for Social Science Research, Copenhagen, Denmark

**Keywords:** social work, child and family welfare, perceived client engagement, questionnaire, caseworker-client relationship

## Abstract

Engagement, defined as the quality of the collaborative relationship between social workers and families, is fundamental to effective statutory social work. A key aspect of worker engagement is how caseworkers perceive client engagement, as these perceptions shape their approach to collaboration, influence decision-making, and ultimately impact service delivery and case outcomes. Understanding and assessing perceived client engagement can help caseworkers strengthen relationships despite the inherent power imbalances and challenges of non-voluntary settings. This paper presents the development of a questionnaire with 23 questions designed to assess caseworker perceptions of client engagement within Denmark’s child and family welfare system. Analysis of responses from 35 caseworkers indicated that most families were perceived as actively engaged and collaborative, with 21 out of 23 questions showing a median score of 4 on a 1–5 scale. Responses for 20 questions covered the full range of response categories, though extreme categories were less frequently used. Interviews with caseworkers suggested that the questionnaire also served as a valuable reflective tool, encouraging them to critically assess their professional relationships and engagement strategies. While initial findings support the questionnaire’s usability, validation studies are needed to evaluate its psychometric properties. Such studies could enhance its value both as a measure of engagement and as a tool for supporting reflective practice in child and family welfare contexts.

## 1 Introduction

The relationship between caseworkers and families is fundamental to effective social work practice. Families involved with social services are often navigating crises, and caseworkers must contend with complexity and uncertainty in their daily work (Engstrom, 2019). Establishing positive and supportive relationships enables caseworkers to assist families in overcoming challenges and improving their overall well-being (Antonopoulou et al., 2024).

Engagement between caseworkers and families is widely recognized as a crucial element in statutory social work, yet there is no universally accepted definition (Cooper Altman, 2008; Mirick, 2014a). Broadly, engagement is understood as the quality of the collaboration between caseworkers and families, akin to the therapeutic alliance between therapists and clients, but occurring within a non-voluntary context (Yatchmenoff, 2005). Yatchmenoff (2005) was one of the first to develop a framework for understanding and measuring client engagement, defining it as the “positive involvement in the helping process” (p. 86). She further delineated engagement into five key dimensions: receptivity, expectancy, investment, mistrust, and the working relationship.

In statutory contexts, engagement is shaped by inherent power imbalances, as caseworkers are responsible for safeguarding children’s well-being, while families may perceive this involvement as intrusive or coercive (Tembo and Studsrød, 2019; Samsonsen and Willumsen, 2015; Bekaert et al., 2021; Toros et al., 2018). Assessing the quality of the relationship is therefore particularly complex and requires recognising engagement as a reciprocal process—dependent not only on parent or service user cooperation, but equally on the caseworker’s active and reflective participation. Yet caseworkers’ perceptions are not neutral; implicit and explicit biases may influence how engagement is assessed and labelled, with consequences for families’ trajectories (Cahalane and Anderson, 2013) Research shows that race, ethnicity, class, and gender can shape subjective assessments of engagement (Rauktis et al., 2011; Miller et al., 2013), with parents described as “uncooperative” or “difficult” based on interpretation rather than behavior (Melz, 2021). This highlights the need for systematic tools that capture caseworker perceptions while acknowledging their socially embedded and potentially biased nature (Melz, 2021).

Overcoming challenges to engagement requires collaborative relationships grounded in mutual trust, shared goals, and active participation from both parties. Core caseworker skills such as empathy, responsiveness to client concerns, and involving parents in planning and decision-making are essential for fostering engagement and achieving positive outcomes (Trotter, 2002; Damiani-Taraba et al., 2017). Other skills—including acknowledging parental efforts, avoiding irrelevant demands, and connecting families with appropriate services—can further strengthen collaboration and predict positive case outcomes (Damiani-Taraba et al., 2017; Gladstone et al., 2014; Gladstone et al., 2012). These skills are especially critical in contested cases or where mistrust and hostility are present (Popoviciu et al., 2013; Ferguson et al., 2021; Fargion and Mauri, 2025). Hostility and distrust should not only be addressed but also recognized as part of the relational dynamic, with reflective and compassionate practice creating space for agency (Ferguson et al., 2021; Fargion and Mauri, 2025). Supporting family participation in CPS decision-making is equally vital for reducing conflict and strengthening collaboration (Merritt, 2020; Seekamp et al., 2023). Together, such competencies form the foundation for building trust and sustaining engagement over time.

These relational processes unfold within the broader welfare systems, which shape the scope, values, and priorities of intervention. In Denmark, the child and family welfare system is rooted in the Nordic Welfare State model (Greve, 2007), integrating both universal and targeted services. It aligns with the ‘child welfare model,’ which prioritizes family engagement, parental strengths, family preservation and early harm prevention, setting a relatively low threshold for intervention before significant harm occurs (Toros et al., 2018; Pösö et al., 2014).

Research links client engagement to positive family experiences (Gladstone et al., 2014; Xu et al., 2017; Estefan et al., 2012) and improved outcomes, such as reduced re-referrals and better service effectiveness (Trotter, 2002; Damiani-Taraba et al., 2017; Cheng and Lo, 2016). Yet engagement is not only determined by family or parent cooperation; caseworker engagement - and in particular caseworker perceptions of client engagement - also play an important role. These perceptions shape how caseworkers interpret collaboration, trust, and family participation, and in turn how they approach collaboration, tailor interventions, ultimately affecting service delivery and case outcomes (Popoviciu et al., 2013). Research suggests that worker perceptions can predict case trajectories and future service involvement, making them especially relevant when working with families facing adversity (Mirick, 2014a; Popoviciu et al., 2013).

Caseworker engagement itself is influenced by factors such as stress, burnout, and professional experience (Gladstone et al., 2014), as well as the degree of family agency and participation in decision-making (Merritt, 2020; Seekamp et al., 2023). These conditions not only affect collaboration strategies but may also shape how caseworkers perceive client engagement. For instance, high levels of stress or burnout can influence whether parental behavior is interpreted as cooperative or resistant.

While caseworker perceptions of client engagement are recognized as important, research on this perspective within mandatory child and family welfare settings remains scarce. Moreover, there is a need to consider how such perceptions may be biased or shaped by systemic inequalities, underscoring the importance of developing measures that can capture these dynamics in nuanced ways (Melz, 2021). This paper is part of a larger project examining both client and worker engagement in child protection services. Here, we focus specifically on the development and pilot testing of a questionnaire designed to assess caseworker perceptions of client engagement within the Danish context. By examining how caseworkers perceive client engagement, this study contributes to a broader understanding of engagement in social work and its practical implications.

## 2 Methods

### 2.1 Development of the questionnaire framework

The Caseworker Perception of Client Engagement Questionnaire (CP-CEQ) was developed to capture caseworkers’ perceptions of client engagement. To ensure coverage of both parents’ general attitudes toward child protective services (CPS) and the relational dynamics between caseworkers and parents, the instrument integrates elements from the Client Engagement Scale (CES) (Yatchmenoff, 2005) and a structured interview guide by Gladstone et al. (2014, 2012). The CP-CEQ was designed for use across all CPS phases—including initial assessment, intervention planning, and follow-up—allowing caseworkers to reflect on engagement at different stages of their involvement with the family.

### 2.2 The client engagement scale (CES)

We first selected 10 questions from the original 37-item CES pool across dimensions of client engagement, while ensuring relevance to the Danish welfare context. These questions primarily assess the parent’s overall engagement with CPS, exploring their willingness to collaborate, trust in the system, and alignment with CPS’s goals and concerns.

The CES was developed by Yatchmenoff (2005) to measure client engagement in child protection services and has been widely applied to explore engagement dynamics (Mirick, 2014a, 2014b; Smith and Pollak, 2020). Based on input from caseworkers, supervisors, and parents who had received services following allegations of abuse or neglect, Yatchmenoff constructed a conceptual framework identifying five key dimensions of client engagement: receptivity, working relationship, expectancy, investment, and mistrust. The original 57-item pool was reduced through feedback from three expert panels to 37 items, and further condensed to a 19-item scale using factor analysis. Items are rated on a 5-point response scale (1 = strongly disagree, 2 = disagree, 3 = not sure, 4 = agree, and 5 = strongly agree), with negatively worded items reverse-scored so that a higher total score indicates greater engagement.

### 2.3 The structured interview guide

To complement the CES-derived questions, we integrated 13 questions from a structured interview guide by Gladstone et al. (2014, 2012), inspired by the CES framework and Trotter’s (Trotter, 2002) work on effective child protection practices. Unlike the CES, which was developed as a client self-report measure, the interview guide was from the outset designed to elicit caseworkers’ perceptions of client engagement. The 13 questions focus on practical and relational dimensions of direct caseworker–parent interactions, including communication, responsiveness, respect, trust, and mutual understanding. Examples include whether the caseworker believes that a parent would reach out for help if needed, whether the parent respects the authority of child welfare services, follows through on agreed actions, listens and stays focused on the issue at hand, or can be trusted to share relevant information. These items thus provide a structured way to capture how caseworkers perceive parents’ willingness and ability to collaborate, extending the focus beyond general attitudes toward CPS as an institution.

### 2.4 The caseworker perception of client engagement questionnaire (CP-CEQ)

The English version of the CP-CEQ is presented in Supplementary Table 1. The CP-CEQ consists of 23 questions assessing caseworkers’ perceptions of client engagement, combining the 10 CES-derived questions with the 13 Gladstone-derived questions. Together, these questions capture both parents’ general engagement with CPS as an institution and the quality of direct interactions between caseworkers and families. All questions are rated on the same 5-point response scale (1 = strongly disagree, 2 = disagree, 3 = not sure, 4 = agree, and 5 = strongly agree), facilitating consistency in scoring. Caseworkers complete a separate CP-CEQ for each family, ensuring that responses reflect engagement on a case-by-case basis.

### 2.5 Translation and cultural adaptation

We followed established procedures for translating and adapting psychological and educational tests (Hambleton, 1995). The process included four key steps: (1) Forward translation into Danish, (2) Expert panel review to ensure conceptual equivalence and clarity, (3) Back-translation into English to verify accuracy, and (4) Pilot testing to identify and resolve any cultural or linguistic issues.

The first author and a researcher with knowledge of Danish social work practice independently translated the 23 questions into Danish. The 10 CES-derived questions were adapted to assess caseworkers’ perceptions of client engagement rather than clients’ self-assessments. Additionally, the midpoint response (3), labelled “not sure,” was revised from “not sure” to “neither agree nor disagree” to reduce ambiguity. To ensure clarity and relevance within the Danish statutory child and family welfare context, two experienced child and family welfare managers reviewed the translation. Their feedback helped refine the wording to align with real-world caseworker practices, ensuring that the questionnaire was both meaningful and applicable. A native English speaker conducted the back-translation, which revealed minor discrepancies that were addressed to improve clarity and precision. Throughout the process, cultural adaptation remained a priority to ensure the questionnaire’s applicability to Danish social work.

As part of the larger project on client and worker engagement in child protection services, we piloted the questionnaire with three caseworkers actively engaged in statutory child and family casework to ensure its suitability for the intended population. Each caseworker completed the questionnaire and provided feedback on its clarity, relevance, and practical applicability. This feedback led to refinements in wording, identification of overlapping questions, and adjustments to questions that appeared too broad or vague.

In addition, we conducted eight individual interviews with social workers from three Danish municipalities. While these interviews primarily explored broader issues of engagement in child protection practice, they also generated useful insights into how caseworkers viewed the questionnaire for assessing perceptions of client engagement, particularly its usability and practical value. Caseworkers generally found the questionnaire relevant and easy to use but emphasized the need for thoughtful application. Some expressed that administrative demands often overshadow relational aspects of their work, and in these cases, they viewed the questionnaire as a useful tool for maintaining focus on collaboration. The interviews also highlighted the questionnaire’s potential as a reflective tool, prompting caseworkers to critically assess the quality of their professional relationships with families. Several caseworkers noted that completing the questionnaire encouraged them to think more deeply about their interactions with families and to identify areas where they could improve engagement strategies. Moreover, some caseworkers suggested that the questionnaire could facilitate a shared understanding between caseworkers and families, potentially enhancing dialog, mutual trust, and collaboration. Further qualitative insights from these interviews, addressing broader aspects of engagement beyond worker perceptions of client engagement, are reported elsewhere (Villumsen et al., 2025).

### 2.6 Field testing the CP-CEQ

The CP-CEQ was field tested within a larger study aimed at developing and testing measures to assessing engagement from both client and caseworker perspectives. A total of 48 caseworkers from four municipalities were invited to participate in the data collection. The four selected municipalities represented a range of population sizes: small (population <30,000), medium (population 30,000–75,000), and larger (population >75,000), ensuring diversity in the sample. Each participating caseworker identified families they were actively working with through child and family welfare services and obtained consent for their participation. Upon receiving consent, the research team received the families’ contact details. All families were then sent an email containing a link to a client engagement questionnaire, which was basically the 37-item CES adapted to Danish.

Caseworkers first completed a background questionnaire on demographic information such as age, education, experience, and municipality. Compassion satisfaction and burnout were assessed using the Professional Quality of Life (PROQOL) (Stamm, 2010), with items rated on a 5-point scale from 1 (Never) to 5 (Very Often). Subscale scores indicate low (below 23), moderate (23–41), and high (42 or higher) levels for both compassion satisfaction and burnout.

Caseworkers received a link to complete the CP-CEQ questionnaire for each family that submitted the client engagement questionnaire. The questions were personalized using the names of the families or parents to ensure relevance and accuracy. Instructions emphasized that responses should be based on the caseworker’s direct experiences with each family. Data were collected through the online platform Defgo, with automated reminders sent after a week, followed by manual follow-ups if necessary.

### 2.7 The sample

Background data was collected from 43 caseworkers, of whom 35 completed the CP-CEQ questionnaire for at least one family. In total, case workers completed the CP-CEQ for 184 questionnaires for individual parents, with each of the case workers completing an average of 5 (range 1–24). Nearly half (n = 17) completed questionnaires for 1–4 families, while 15 completed 5–9. Only three caseworkers completed questionnaires for 10, 11, and 24 families, respectively. To mitigate potential biases arising from multiple responses from the same caseworker, we randomly selected one response per caseworker for analysis, resulting in a final sample of 35 unique responses.

Characteristics of the 35 caseworkers are presented in Table 1.

**Table 1:** Demographic, professional, and case characteristics of the 35 caseworkers.

		*N*	%
Municipality	Large	27	77
	Small A	3	9
	Small B	5	14
Gender	Female	31	89
	Male	2	6
	Not disclosed	2	6
Education	Social worker	29	83
	Other	6	17
Experience within child welfare services	0–2 years	7	20
	3–5 years	5	14
	5 + years	23	66
Participant or parents born outside Denmark	Yes	4	11
	No	31	89
Severity of case	Not severe	3	9
	Less severe	11	31
	Moderately severe	17	49
	Very severe	4	11
Compassion satisfaction	High	14	40
	Medium	20	57
	Low	1	3
Burnout	High	0	0
	Medium	9	26
	Low	26	74

Population: small (population <30,000), medium (population 30,000–75,000), and larger (population >75,000).

The majority of the 35 caseworkers were female and trained social workers, mostly based in the larger municipality. Most had over five years of experience in child protection and had worked with their assigned families for more than two years. Cases were mainly rated as ‘Moderately severe’ (49%) or ‘Less severe’ (31%), with a smaller proportion rated as ‘Very severe’ (11%) or ‘Not severe’ (9%). Regarding professional well-being most reported medium or high compassion, and low levels of burnout. This distribution highlights the diverse challenges encountered within their caseloads.

## 3 Results

As a simple evaluation of the usability of the CP-CEQ in the intended study population, we conducted a descriptive analysis of the responses of the 35 caseworkers. Table 2 shows the frequency distribution, median, and mode for each of the 23 questions.

**Table 2:** Frequency distribution of responses for the CP-CEQ.

Question number	Worker perception of client engagement questionnaire (CP-CEQ)	Strongly disagree	Disagree	Neither agree nor disagree	Agree	Strongly agree	Median	Mode
1	I think (name of parent) really wants to make use of the services that CPS is providing her/him.	1	3	3	15	13	4	4
2	I think (name of parent) finds it difficult to work with me.	6	13	12	3	1	4	4R
3	I think (name of parent) believes that CPS will use what s/he says to make her/him look bad.	6	15	11	1	2	4	4R
4	I think (name of parent) is not just going through the motions. S/he is really involved in working with CPS.	4	2	4	16	9	4	4
5	I think (name of parent) shares the same concerns that CPS has for her/his children.	1	4	4	21	5	4	4
6	I think (name of parent) would say that s/he and I agree about what is best for her/his child.	1	3	6	21	4	4	4
7	I think (name of parent) feels that s/he can trust CPS to be fair and to see her/his side of things.	1	6	6	19	3	4	4
8	I think (name of parent) would say that what CPS wants her/him to do is the same as what s/he wants.	1	6	7	18	3	4	4
9	I think (name of parent) would say that there were definitely some problems in her/his family that CPS saw.	2	4	18	11	0	3	3
10	I think (name of parent) would say that CPS is helping her/his family get stronger.	3	2	14	13	3	3	3
11	I think (name of parent) is able to provide effective care to his/her child(ren).	3	4	6	15	7	4	4
12	I think (name of parent) would call me if s/he needed assistance with her/his child.	1	0	8	17	9	4	4
13	I think (name of parent) would tell me things about her/his child(ren) that I need to know.	0	2	6	18	9	4	4
14	I think that (name of parent) respects the expertise and authority of the CPS.	1	3	6	19	6	4	4
15	I think (name of parent) and I are working toward the same goals.	1	4	3	20	7	4	4
16	I think (name of parent) is able to listen to me.	1	1	3	25	5	4	4
17	I think (name of parent) follows up on things her/him to do.	1	4	5	18	7	4	4
18	I think that (name of parent) is able to focus on the issue at hand rather than go off on tangents.	2	3	10	15	5	4	4
19	I feel like (name of parent) can see my side of things.	1	3	11	18	2	4	4
20	I find that (name of parent) is friendly and easy to talk to.	0	1	3	18	13	4	4
21	I think that (name of parent) trusts me.	1	1	11	18	4	4	4
22	I think that (name of parent) understands why CPS is involved with her/his family.	1	5	2	19	8	4	4
23	I believe (name of parent) is only telling me things s/he thinks I want to hear.	4	19	7	4	1	4	4R

CPS, child protective services. R, reverse scored.

For 20 out of 23 questions responses spanned the full range of categories. However, for three questions one of the most extreme response categories were not used. For question 8 [*‘I think (name of parent) would say that there were definitely some problems in her/his family that CPS saw’*], none of the 35 caseworkers endorsed the most positive response category, ‘strongly agree’. Conversely, the most negative category ‘strongly disagree’, was not endorsed for question 13 [*‘I think (name of parent) would tell me things about her/his child(ren) that I need to know’*]or question 20 [*‘I find that (name of parent) is friendly and easy to talk to’*]. Additionally, three other questions rarely received “disagree” or “strongly disagree” responses (with only two or fewer caseworkers selecting these categories). These questions were: 12: *‘I think (name of parent) would call me if s/he needed assistance with her/his child’*, 16: *‘I think (name of parent) is able to listen to me’*, and 21: *‘I think that (name of parent) trusts me’*. Notably, all of these questions were derived from the interview questions by Gladstone et al. (2014, 2012).

The median and mode scores for 21 out of 23 questions were both 4 on a 1–5 scale, where higher scores indicate more positive perceptions of clients’ engagement. These results suggest that caseworkers generally perceived most families as actively engaged and collaborative. For the remaining two questions [question 9: *‘I think (name of parent) would say that there were definitely some problems in her/his family that CPS saw’* and question 10: *‘I think (name of parent) would say that CPS is helping her/his family get stronger’*], the median and mode scores were 3. This suggests a slightly more neutral perception of the parent’s engagement for these two questions.

## 4 Discussion

This study is part of a larger project examining engagement in child protection services from both caseworker and client perspectives. Here, we specifically introduced and piloted a questionnaire with 23 questions designed to assess caseworkers’ perceptions of client engagement within Denmark’s child and family welfare system. The analysis of responses from 35 caseworkers indicated that most questions were rated positively, suggesting that families were generally perceived as actively engaged and collaborative. For 21 out of 23 questions, the median score was 4 on a 1–5 scale, suggesting that families were typically seen as actively engaged and collaborative. Additionally, responses for 20 out of 23 questions covered the full range of response categories, though the most extreme categories (‘strongly agree’ and ‘strongly disagree’) were used less frequently.

Interestingly, the five questions where extreme response categories were rarely used all were derived from the interview questions from Gladstone et al. (2014, 2012). This may suggest that caseworkers tend to rate families more positively on practical and relational aspects of engagement, emphasizing the quality of their direct interactions with parents.

Taking the small sample of 35 caseworkers into account, the results of our initial descriptive analysis do not raise immediate concerns regarding the questionnaire’s usability or appropriateness for the intended population. However, proper psychometric validity studies with sufficiently large and independent samples of caseworkers is necessary to rigorously assess its measurement properties, including invariance across subgroups. Such studies would disclose its psychometric properties and show whether further refinements are needed to precise and generalizable insights into caseworker perceptions of client engagement.

The interviews with caseworkers highlighted an additional, valuable function of the questionnaire beyond its role as a standardized measure of engagement. Caseworkers reported that completing the questionnaire prompted them to reflect more deeply on their professional relationships and engagement strategies. This reflective process was perceived as beneficial for maintaining a focus on collaboration, which can often be overshadowed by the administrative demands of statutory social work. The questionnaire’s ability to prompt reflection also suggests potential for use in supervision or other forms of reflective practice, where it may facilitate shared learning and ongoing professional development. While not explored in this study, such applications could be relevant at both individual and organizational levels.

Additionally, this reflective aspect may help bridge different perspectives on engagement between caseworkers and families, potentially enhancing dialog and mutual understanding. Several caseworkers noted that the questionnaire provided a structured opportunity to consider relational dynamics and to identify areas for improvement in their interactions with families. Used together with the CES completed by parents, the questionnaire may support more balanced dialog and reduce power asymmetries. This dual function suggests that the questionnaire could serve both as a measure of caseworker perceptions of client engagement and as a practical tool for supporting reflective practice.

As part of the broader project, this paper focuses on caseworker perceptions of client engagement as a first step toward capturing engagement as a relational, two-sided process. With validation and refinement, the CP-CEQ may serve as both a measure of caseworker perceptions of client engagement and as a practical tool for enhancing collaboration and relational quality in child and family welfare contexts.

### 4.1 Limitations and future directions

The main limitation of this study is the small and self-selected sample of 35 caseworkers, which may not adequately represent the broader population of social workers. Participants were likely more confident in their professional relationships, potentially introducing a selection bias. Additionally, the caseworker perceptions of client engagement questionnaire require validity studies to assess its effectiveness and identify potential areas for refinement. This is important both as a standalone tool and in combination with the client engagement scale completed by families.

Future research should aim to expand the sample to include caseworkers with a wider range of professional backgrounds, experience levels, and case complexities to facilitate a comprehensive psychometric validation of the questionnaire. Such studies should utilize truly independent data—meaning one case per caseworker—to avoid introducing dispositional bias. An additional direction for future research would be to examine the level of agreement between parents and caseworkers on the 10 CES-derived questions, for which paired responses have been collected. This could provide a more nuanced understanding of engagement perceptions from both perspectives.

Despite these limitations, the findings suggest that the CP-CEQ holds promise as a tool for supporting professional reflection and enhancing collaborative dynamics in statutory social work. With validation and potential refinements, it has the potential to provide valuable insights into worker-client relationships and contribute to improving engagement and service delivery within child welfare contexts.

## Data Availability

To protect participant privacy, the data generated and analyzed during this study are not publicly available. Deidentified data may be made available to qualified researchers upon reasonable request, subject to institutional review and data use agreements. Requests should be directed to the corresponding author.
