# Supplementary material for: Assessing caseworker perceptions of client engagement in Danish child and family welfare
Source: Front Sociol. 2025 Oct 31;10:1591213. doi: 10.3389/fsoc.2025.1591213 (PMC12616554; doi:10.3389/fsoc.2025.1591213)
Supplement: Supplementary file 1 [file Table_1.DOCX]

Supplementary Table 1 English version of the CP-CEQ

|  | English | Origin | |
| --- | --- | --- | --- |
| 1 | I think (name of parent) really wants to make use of the services that CPS is providing her/him. | 5 | INV |
| 2R | I think (name of parent) finds it difficult to work with me. | 6 | WR |
| 3R | I think (name of parent) believes that CPS will use what s/he says to make her/him look bad. | 7 | MIS |
| 4 | I think (name of parent) is not just going through the motions. S/he is really involved in working with CPS. | 14 | INV |
| 5 | I think (name of parent) shares the same concerns that CPS has for her/his children. | 15 | REC |
| 6 | I think (name of parent) would say that s/he and I agree about what is best for her/his child. | 16 | WR |
| 7 | I think (name of parent) feels that s/he can trust CPS to be fair and to see her/his side of things. | 18 | MIS |
| 8 | I think (name of parent) would say that what CPS wants her/him to do is the same as what s/he wants. | 22 | INV |
| 9 | I think (name of parent) would say that there were definitely some problems in her/his family that CPS saw. | 24 | REC |
| 10 | I think (name of parent) would say that CPS is helping her/his family get stronger. | 36 | EXP |
| 11 | I think (name of parent) is able to provide effective care to his/her child(ren). |  | INT |
| 12 | I think (name of parent) would call me if s/he needed assistance with her/his child. |  | INT |
| 13 | I think (name of parent) would tell me things about her/his child(ren) that I need to know. |  | INT |
| 14 | I think that (name of parent) respects the expertise and authority of the CPS. |  | INT |
| 15 | I think (name of parent) and I are working toward the same goals. |  | INT |
| 16 | I think (name of parent) is able to listen to me. |  | INT |
| 17 | I think (name of parent) follows up on things her/him to do. |  | INT |
| 18 | I think that (name of parent) is able to focus on the issue at hand rather than go off on tangents. |  | INT |
| 19 | I feel like (name of parent) can see my side of things. |  | INT |
| 20 | I find that (name of parent) is friendly and easy to talk to. |  | INT |
| 21 | I think that (name of parent) trusts me. |  | INT |
| 22 | I think that (name of parent) understands why CPS is involved with her/his family. |  | INT |
| 23R | I believe (name of parent) is only telling me things s/he thinks I want to hear. |  | INT |

Notes: CPS: Child Protective Services. R: reverse scored. Origin refers to item number and subscale in the 37-item Client Engagement Scale (CES): INV: investment, WR: working relationship, MIS: mistrust, REC: Receptivity; EXP: Expectancy, INT: from the Structured Interview Guide by Gladstone et al.
